# New types of investments needed to address barriers to scaling up wildfire risk mitigation

**DOI:** 10.1186/s42408-022-00155-2

**Published:** 2022-12-13

**Authors:** Laurie Yung, Benjamin J. Gray, Carina Wyborn, Brett Alan Miller, Daniel R. Williams, Maureen Essen

**Affiliations:** 1 Department of Society and Conservation, W.A. Franke College of Forestry and Conservation, Missoula, MT 59812 USA; 2 USDA Forest Service, Willows, CA 95988 USA; 3 Institute for Water Futures, Fenner School of Environment and Society, The Australian National University, Canberra, ACT 2601 Australia; 4 USDA Forest Service, Rocky Mountain Research Station, 240 West Prospect Road, Fort Collins, CO 80526 USA; 5 USDA Forest Service, Region One, 26 Fort Missoula Road, Missoula, MT 59804 USA

**Keywords:** Fuel treatment, Natural resource collaboration, Wildfire governance, Wildfire planning and policy, Wildfire risk mitigation, Wildfire social science

## Abstract

**Background:**

Wildfire mitigation is becoming increasingly urgent, but despite the availability of mitigation tools, such as prescribed fire, managed wildfire, and mechanical thinning, the USA has been unable to scale up mitigation. Limited agency capacity, inability to work across jurisdictions, lack of public support, and procedural delays have all been cited as barriers to mitigation. But in the context of limited resources and increasing urgency, how should agencies prioritize investments to address these barriers?

**Results:**

To better understand different investments for scaling up mitigation, we examined how the wildfire problem is framed, building on existing social science demonstrating that agency approaches depend in part on how problems are framed. Using national-level policy documents and in-depth interviews, we found three ways of framing the barriers to scaling up mitigation, each emphasizing certain aspects of the problem and prioritizing different solutions or investments. The first framing, the *Usual Suspects*, focused on inadequate resources, cumbersome procedural requirements, delays due to litigation, and lack of public support. The solutions—to increase funding, streamline NEPA, limit litigation, and educate the public—suggest that more resources and fewer restrictions will enable agencies to scale up mitigation. The second framing, *Agency-Agency Partnerships*, focused on the ways that organizational structure and capacity constrain the development of effective cross-boundary collaboration. Here solutions prioritized organizational changes and capacity building to enable agencies to navigate different missions and build trust in order to develop shared priorities. The third framing, *Engaging the Public*, focused on lack of public support for mitigation, the need for meaningful public engagement and multi-stakeholder collaboration, and investments to build support to scale up mitigation.

**Conclusions:**

This analysis reveals that investing in collaborative capacity to advance agency-agency partnerships and public engagement might not slow down mitigation, but rather enable agencies to “go slow to go fast” by building the support and mechanisms necessary to increase the pace and scale of mitigation work. Reframing the wildfire problem through a careful analysis of competing frames and the underlying assumptions that privilege particular solutions can reveal a broader suite of solutions that address the range of key barriers.

## Resumen

**Antecedentes:**

La mitigación de incendios es una tarea urgente, aunque a pesar de la disponibilidad de herramientas de mitigación, tales como quemas prescriptas, manejo del fuego, y raleos mecánicos, EEUU no ha sido aún capaz de aumentar proporcionalmente la mitigación de sus incendios. Las limitaciones en las capacidades de las agencias, la inhabilidad de trabajar entre jurisdicciones, la falta de apoyo de la sociedad, y demoras en los procedimientos, han sido citados como barreras para la mitigación. Ahora bien, en el contexto de recursos limitados e incrementos en las urgencias, ¿cómo deberían las agencias priorizar las inversiones para enfrentar esas barreras?

**Resultados:**

Para entender mejor las distintas inversiones para aumentar proporcionalmente las tareas de mitigación, examinamos cómo el problema de los incendios es enmarcado y construido en la ciencia social existente, demostrando que los enfoques de las agencias dependen, en parte, en cómo esos problemas son abordados. Usando documentos de políticas públicas y entrevistas profundas e intuitivas, encontramos tres vías de enmarcar las barreras para aumentar proporcionalmente la mitigación, cada una enfocando ciertos aspectos del problema y priorizando diferentes soluciones o inversiones. El primer enfoque, las “Sospechas Habituales”, estuvo orientado hacia la inadecuación de los recursos, procedimientos incómodos, demoras debidas a litigios, y la falta de respaldo público. Las soluciones -incrementar los recursos, simplificar los procedimientos de la ley ambiental, limitar los litigios, y educar al público- sugieren que más recursos y menos restricciones permitirán a las agencias aumentar proporcionalmente la mitigación. El segundo enfoque “Asociación Agencia-Agencia”, se orientó en la forma en que la estructura organizacional y su capacidad condicionan el desarrollo de una colaboración efectiva entre agencias. La solución aquí prioriza los cambios organizacionales y la capacidad de construcción para permitir a las agencias acometer diferentes misiones y crear confianza para poder desarrollar prioridades compartidas. El tercer enfoque “Comprometer al Público” se enfoca en la falta de apoyo del público para la mitigación, y la necesidad de atraer al público y la colaboración de diferentes ciudadanos interesados, en realizar aportes necesarios para construir aportes para el desarrollo de prioridades de mitigación.

**Conclusiones:**

Este análisis revela que la inversión en capacidad colaborativa para avanzar en la asociación agencia-agencia y el compromiso público puede no reducir la mitigación, más sin embargo permitir a las agencias “ir despacio para ir más rápido” para construir el soporte y mecanismos necesarios para incrementar la velocidad y la escala del trabajo de mitigación. Reencuadrar el problema de los incendios forestales a través de un cuidadoso análisis de encajes competitivos y suposiciones subyacentes que privilegie soluciones particulares, puede revelar un más amplio conjunto de soluciones que atienda el rango de barreras claves.

## Background

On January 18, 2022, the USDA Forest Service announced a 10-year plan to reduce wildfire “risk to people, communities, natural resources, and other values at the scale of wildfire risk” by treating an additional 20 million acres of National Forest System land and 30 million acres of other “Federal, State, Tribal, and private lands in the west” by 2032 (USDA 2022:3-4). This ambitious plan is in response to fire seasons becoming longer, drier, and more intense, in part due to climate change (Abatzoglou and Williams 2016). Longer fire seasons and larger fires have led to more smoke, which has contributed to increasing PM2.5 (fine particulate matter) exposure in the Western US (Burke et al. 2020). Further, approximately 50 million US homes are now in the wildland-urban interface, with 1 million added every 3 years (Burke et al. 2020), putting an increasing amount of infrastructure at risk from wildland fire. The economic and health impacts of wildland fires are projected to worsen and the cost of fire suppression has skyrocketed (Bowman et al. 2020, Burke et al. 2020, Roman et al. 2020).

While a number of wildfire risk mitigation tools are available, including prescribed fire, managed wildfire, and mechanical thinning, there has been an “under-investment” in mitigation, especially “given the massive overall growth in wildfire risk” (Burke et al. 2020:2). Part of this needed investment is monetary, which is why the Forest Service is planning to spend $2.42 billion for fuels related projects through 2026 (Gabbert 2022). But the barriers to scaling up wildfire risk mitigation are more than financial. For instance, prescribed fire is regarded as a highly effective and relatively inexpensive tool for mitigating wildfire risk, but the use of prescribed fire has not increased over the last 20 years (Kolden 2019). And despite the benefits of managed wildfire, more than 95% of wildfires are suppressed (USDA 2015). Additionally, scaling up mitigation means more than increasing the number of acres treated, because the impact of mitigation depends on the location and type of treatment (Charnley et al. 2015).

Scaling up mitigation can be challenging because it often requires substantial collaboration across agencies and ownerships to implement at a landscape scale, especially in the case of prescribed fire (Quinn-Davidson and Varner 2012). Other barriers to prescribed fire include lack of capacity and inadequate funding, lack of agency direction, political conflict, lack of public support, air quality regulations and narrow burn windows, environmental laws, and liability issues (Miller et al. 2022, Schultz et al. 2019a, Quinn-Davidson and Varner 2012, Cleaves et al. 2000). Proposed mitigation projects can also be controversial and agencies often struggle to advance proposals that meet the needs of various groups with competing values and interests (Mylek and Schirmer 2020, Ostergren et al. 2006). Further, fuel treatments that focus on the wildland-urban interface are much more visible and thus subject to increased public scrutiny (Brenkert-Smith et al. 2019).

Public confidence in agencies and perceptions of manager competence to implement fuel treatment are associated with increased social acceptability of smoke, prescribed fire, and mechanical thinning (Toman et al. 2011, Olsen et al. 2017). People who are familiar with forest conditions and fuel treatment techniques are more likely to trust agencies and support proposed mitigation actions (Toman et al. 2013, McCaffrey et al. 2013, Diaz et al. 2016). And residents in the wildland urban interface specifically prefer active management to reduce fuels over no action (Toman et al. 2013). However, numerous studies indicate that public outreach and education alone are not sufficient to increase support for specific fuel treatment proposals (Brunson and Shindler 2004, McCaffrey 2004, McCaffrey and Olsen 2012, Brenkert-Smith et al. 2017, Dupéy and Smith 2018, Wilson et al. 2018).

Public support for proposed fuel treatments is highly contingent on relationships between the public and agencies, specifically trust in those agencies, which can be improved through long-term, meaningful engagement with the public (Vaske et al. 2007, Brenkert-Smith 2010, Olsen 2010, Toman et al. 2011, McCaffrey et al. 2013, Toman et al. 2014, Olsen et al. 2017). More specifically, formal collaboratives, informal interactions, and especially early public involvement through open and transparent decision-making processes increase public trust (McCaffrey et al. 2013). However, public engagement only builds trust in agencies when the public views these opportunities as meaningful and when agencies clearly take public input into account in decision-making (Olsen 2010, Olsen and Sharp 2013).

In the absence of a shared understanding of the fire problem, Brenkert-Smith et al. (2019) suggest that public opposition and litigation will continue to delay and prevent implementation of mitigation projects. In fact, agencies have long-argued that appeals and lawsuits, along with the planning and analysis mandated by the National Environmental Policy Act (NEPA), have prevented them from addressing wildfire risk, concerns that led to limits on administrative appeals in the 2003 Healthy Forests Restoration Act (Ostergren et al. 2006). Interestingly, while there is public support for streamlining appeals, litigation, and environmental review, to speed the implementation of fuel treatments, public support is even stronger for more meaningful involvement of local communities and residents in the planning and decision-making process around mitigation (Ostergren et al. 2006).

Given the myriad barriers that have been identified, how should government agencies prioritize their limited resources? Which investments can effectively scale up mitigation work? According to Crow et al. (2017a), agency approaches to natural hazards like wildfire depend in part on the way in which narratives frame a problem and advance a particular solution. More specifically, how a problem is framed can influence the types of solutions that are pursued (Crow et al. 2017b, Stone 1989). As such, understanding how the problem of wildfire is framed is critical to identifying the suite of solutions being advanced. Frames help us organize and understand the world, allowing us to shape the problems we encounter (Gray 2003). According to Gray (2003), “frames are used to (1) define issues, (2) shape what action should be taken and by whom, (3) protect oneself, (4) justify a stance we are taking on an issue, and (5) mobilize people to take or refrain from action on issues.”

Framing involves discarding or devaluing information that does not fit or align with the dominant or preferred frame (Elliott et al. 2003). Through this process, frames are used to sift through information and determine which elements of a particular problem are most important (Nisbet 2009). Thus, each frame highlights some dimensions of a problem as more relevant than others (Nisbet 2009). Because frames direct our attention to some aspects of reality while obscuring other aspects, they promote specific ways of conceptualizing problems and solutions, and thus privilege certain values and outcomes (Yung et al. 2013). As a result, how one sees and defines a particular problem has a profound effect on the strategies and actions for addressing that problem, because problem frames embody the core assumptions about how to approach the problem (Bardwell 1991). Put simply, the way that we define or frame the problem shapes policy and practice (Collins and Ison 2009). More specifically, “frames…set a specific train of thought in motion, communicating why an issue might be a problem, who or what might be responsible for it, and what should be done about it” (Nisbet 2009). In the arena of wildland fire, Crow et al. (2017a) found that different problem definitions were associated with different solutions. In their analysis of Forest Service wildfire management, Schultz et al. (2019b:3) also suggest that “problem definition plays an important role in both structuring decisions and framing solutions.”

Because particular ways of framing a problem emphasize or highlight some ideas while excluding others (Druckman 2001), a dominant frame can constrain political debate and limit policy options by implying that a particular way of defining the problem is more legitimate (Jasanoff and Wynne 1998). In the context of federal lands, frames can privilege and legitimate the views and interests of particular groups over others (Yung et al. 2010). At the same time, competing frames can both explain and drive conflict over the management of federal lands (Klyza 1991). Moreover, the way in which a problem like wildfire is framed can influence who participates in decision-making processes and the specific projects that are prioritized (Williams et al. 2012).

People often take the assumptions that underlie a particular frame for granted, rarely questioning or examining them (Cairney and Kwiatkowski 2017). However, if different ways of framing a problem are explicated, this process can expand people’s understanding of the problem and enable them to consider a broader range of solutions (Bardwell 1991, Gray 2003). The process of “reframing” requires reflecting on different ways of conceptualizing a problem (Elliott et al. 2003). The findings described below help advance this type of analysis and reflection, by describing three different ways of framing the wildfire problem, and specifically the barriers to scaling up mitigation. Not surprisingly, these three problem framings were aligned with specific sets of solutions and investments, and reveal important tensions between different approaches to advancing mitigation.

## Methods

### Study sites

This research is part of the Co-Managing Fire Risk Transmission (CoMFRT) partnership, a long-term, multi-site research project focused on understanding approaches to managing wildfire risk across boundaries and at landscape scales, and how agencies, communities, tribes, and other stakeholders work together to reduce risk. To better understand barriers to scaling up mitigation, we examined national-level policy documents and conducted in-depth interviews with wildfire actors working in North Central Washington and Northern Utah, and at regional and national scales in the USA. The Washington and Utah study sites were selected because both have recently experienced large wildfires, have extensive wildland-urban interface development, and have developed risk reduction measures intended to address wildfire risk.

North Central Washington encompasses the eastern foothills of the Cascade Range, just a few hours from the population centers of Seattle and Spokane. Wenatchee (pop. 34,000) is the largest community in North Central Washington and the county seat for Chelan County (population of about 72,000 people). Eighty-one percent of Chelan County is publicly owned (State of Washington 1999), the bulk of which is the Okanogan-Wenatchee National Forest. North Central Washington has experienced significant growth and amenity-based in-migration, spurring further development of wildland-urban interface areas.

The Northern Utah study site includes the eight counties that surround the Wasatch Mountains, where approximately 80% of Utah’s population lives. From Ogden to Provo, with the capital of Salt Lake City in between, the Wasatch Front is the most densely populated part of Northern Utah. Residential development is occurring in foothills and canyons with convenient access to the larger metro area, increasing the amount of infrastructure in close proximity to the National Forest lands.

### Data collection and analysis

This study employed a mixed methods approach that included in-depth, semi-structured interviews (Hesse-Biber and Leavy 2006) and document analysis. We conducted 62 in-depth, semi-structured interviews with federal and state agency, local government, NGO staff, and tribes (see Table 1). For 42 interviews, an initial list of interviewees was developed through CoMFRT meetings and contacts in Wenatchee, Salt Lake City, and Washington D.C. and expanded through chain referral (Brandenburg and Carrol 1995). Purposive sampling was utilized to ensure that a range of wildfire actors and perspectives were included in the research (Patterson and Williams 2002). An additional 20 interviews were drawn from a social network analysis (SNA) from Washington conducted by researchers at Portland State University (Nielsen-Pincus et al., 2019). Due to the COVID-19 pandemic, we were unable to conduct the additional SNA interviews in Utah. Further, despite the importance of tribes, cultural burning, and traditional ecological knowledge, we were only able to interview two tribal members.

**Table 1:** Sample characteristics

	Regional or National	Utah	Washington	Total
Federal agencies	2	3	11	16
State agencies		6	8	14
Tribes			2	2
Local agencies		5	11	16
NGOs	2		12	14
Total	4	14	44	62

An interview guide was utilized to ensure comparability across interviews and also allow for emergent phenomena (Hesse-Biber and Leavy 2006, Patterson and Williams 2002). Interview questions focused on approaches to working across boundaries and scales, as well as opportunities and challenges to scaling up mitigation work. SNA interviews also included questions about the network and collaboration.

To better understand how barriers to scaling up mitigation were conceptualized at the national level, we analyzed two documents, the National Cohesive Wildland Fire Management Strategy (the Cohesive Strategy) and the Cohesive Strategy Crosswalk and Strategic Alignment (the Crosswalk). The Cohesive Strategy, completed by the Departments of Interior and Agriculture in 2010 and revised most recently in 2014, seeks to advance collaboration across agencies, organizations, communities, and landscapes to realize three goals: resilient landscapes, fire-adapted communities, and safe and effective wildfire response (The National Strategy 2014). Conducted in 2016 by the National Strategic Committee, the Crosswalk reviewed progress under the Cohesive Strategy, providing a detailed evaluation of wildland fire work, with an inventory of opportunities and barriers.

The interviews were recorded, professionally transcribed, and analyzed using NVivo 11 software. Both interviews and policy documents were coded through an iterative process that enabled a dialogue between the data and relevant social theory (Layder 1998). Initial coding was conducted by four of the authors, used a thematic approach, and focused on emergent phenomena (Rubin and Rubin 2005). Codes were then refined based on existing literature and social theory. The mixed methods approach, using both interviews and policy documents, enabled us to iterate between the two data sources during analysis. More specifically, the interviews provided an understanding of how a range of wildfire actors at various scales frame barriers, whereas the two policy documents enabled us to identify official, national-level framings about barriers. In combination, these two data sources provide insight into how the barriers are framed and which specific solutions are advanced under different framings. After codes were refined and coding completed, the author team discussed the findings and outlined this manuscript, focused on the three framings described below in the results. To ensure intercoder reliability, the lead author then went through all of the data that was potentially relevant to this manuscript, recoded it, and organized it into the three frames below. Once the data was organized according to this framework, the author team determined that the analysis was sufficient to move to the writing stage of the process.

## Results

Our analysis found three different ways of framing the barriers to scaling up mitigation, each emphasizing certain aspects of the problem and prioritizing certain types of solutions. Most of the people we interviewed emphasized only one of these three frames, but there were a handful of interviewees who focused on more than one.

### The Usual Suspects: commonly cited barriers to scaling up mitigation

The *Usual Suspects* is a framing that tends to dominate the discussion in both the Crosswalk and our interviews. This framing focuses on the following barriers: (1) lack of resources, especially funding; (2) cumbersome and time-consuming policy and procedural requirements; (3) litigation that halts or delays implementation; and (4) lack of a shared understanding of the problem and solutions among the public.

Not surprisingly, lack of resources (particularly funding) emerged as a significant barrier. The Crosswalk states that “funding for fuels treatments is scarce among all entities” (Appendix F6). Interviewees in both Washington and Utah frequently discussed lack of funding and lack of capacity, stating that existing resources are “not enough to keep pace” with wildfire risk and that lack of funding “is always a real challenge…a significant challenge.” And despite an interest in sharing resources to build capacity, the current system for sharing resources for mitigation work, particularly for prescribed burning, was described by the Crosswalk as “inefficient and cumbersome” in comparison with the system for sharing resources for wildfire response and suppression (Appendix F2-F3). Interviewees agreed, saying that the mechanisms for billing across agencies that exist for incident response do not exist for mitigation. They explained that “there’s capacity in one place that another organization could use,” but these barriers often stymie efforts to share those resources. For example, as a tribal member explained, it can be difficult to compensate tribal employees for conducting cultural resource surveys on federal land in advance of prescribed burning. Further, while the recently expanded Good Neighbor Authority (GNA) includes mechanisms for sharing capacity across agencies, challenges to using GNA remain (Crosswalk Appendix F5). For example, one Forest Service employee pointed out that the federal agencies could not, at the time, turn prescribed burning over to Oregon and Washington state agencies because state employees lacked the required training. Where barriers were framed as lack of capacity for implementation, the solutions cited included increased funding to treat more acres and better mechanisms for sharing resources and building capacity across agencies.

Policy was also regarded as a significant barrier. The Crosswalk states that “landscape scale restoration is often difficult to achieve due to the complex procedural requirements of federal laws, rules, and policies” (Appendix E5) and that:

Years can pass before collaboratively developed and approved projects can be implemented due to the various planning requirements and processes, and allocation of resources necessary to allow the projects to move forward. Ability to implement such projects on the ground in a timely manner should be increased. (Appendix F7-F8)

A local Fire Chief from Utah echoed this concern, suggesting that federal regulations do not allow for some types of mitigation work, saying “so many people would love to harvest those dead trees or just at least get rid of them but I know federal regulations, they don’t allow that. They have a lot of other red tape that they have to cut through.” Similarly, a Forest Service employee cited the planning processes as “one of the largest barriers” because of “competing values” and the challenges of getting “buy-in from the biologists and other specialists.”

Interviewees talked extensively about the lengthy environmental impact assessments required by the regulations that implement the National Environmental Policy Act (NEPA), arguing that the NEPA process is expensive and delays implementation of fuel reduction projects. Interviewees described NEPA as a “headache,” “one of our bigger frustrations,” and an impediment to collaboration between state and federal agencies. A Utah state agency employee described how federal policy requirements made it difficult to collaborate with federal agencies on large-scale treatments, saying:

We’re ready to go. And we can really implement something in a matter of months. And we’ve got to wait two years for the Forest Service to figure it out. Definitely a big headache, right? So, you move at the speed of your slowest person. So you have to shelf that idea, maybe, if we really want to collaborate.

One County employee complained that NEPA is a “pretty open door on objections” that “encourages too much engagement, too much participation.”

While NEPA was characterized as the primary policy barrier, air quality rules were seen as restricting prescribed fire in both the Crosswalk (appendix E3) and among our interviewees, who explained that exceeding “parameters on particulate matter” was a “real challenge” and meant shutting down prescribed fires. Interviewees also perceived the Endangered Species Act as a barrier to doing fuels work in areas with threatened or endangered species.

When policy was highlighted as the barrier, interviewees and the Crosswalk converged on a similar solution, to streamline policy requirements, in particular NEPA. The Crosswalk focuses specifically on the need for “actions that break down identified regulatory, process, and other administrative barriers and address critical success factors” (p 18) and calls for federal agencies “to expedite the planning/collaboration process to treat large landscapes” (CSF14-2) and to “seek relief from impediments in the Forest Service Planning Rule for fuels management” (CSF14-5). According to the Crosswalk, “Alternative federal processes must allow more efficient and less costly processes that decrease the time needed for the necessary planning, and aid in the development of solutions promoting large scale active management and fuels treatments” (Appendix F7-F8). Interviewees, including a Utah state employee, argued that the federal government should “speed up that process.”

Litigation was also regarded as a major barrier. According to the Crosswalk (Appendix F7-F8):

Federal laws and regulations, particularly the National Environmental Policy Act (NEPA), the Endangered Species Act (ESA), and the Equal Access to Justice Act (EAJA), have been used by groups…to limit or delay the implementation of these projects…This will likely require new legislation or the modification of current legislation in order to allow collaborative, outcome-based solutions to withstand judicial challenges.

In addition, fear of litigation was believed to influence the types of projects that are proposed. One timber industry representative argued that the Forest Service is “afraid to sneeze without getting litigated, and they’ve had most of their tools taken away from them, or tied up so heavily in litigation that they just can't get business back.” One federal wildfire leader suggested that it is more fear of lawsuits than actual lawsuits, saying “you get this fear of lawsuits, and I think what they found is, yes in certain areas it’s very well founded, but in most areas it’s somewhat unfounded, and there’s not, percentage wise, as many of those plans that get challenged as we often would think.” The Crosswalk suggests that “new interpretation and engagement with key partners can take advantage of flexibility that currently exists, but may not be exercised for fear of litigation” (Appendix E5). In short, litigation was characterized as both delaying implementation and influencing the types of projects proposed. Thus, limiting litigation was seen as a way to address that barrier.

The fourth commonly discussed “usual suspects” barrier was lack of public understanding. A state agency employee summed up a sentiment echoed by many interviewees, saying that “increasing public awareness and understanding” was “critical.” According to a Forest Service employee:

We have a lot of members of our publics, people who live in our communities who have a very good, very nuanced understanding of fire’s role on landscapes. And we have a lot of other people that don’t have any context for it at all. And all they see is fire is bad…So, now we have conflict, this difference in the worldview about what the challenges are, and what reasonable solutions are.

This NGO employee described “misunderstandings,” saying:

I’ve heard crazy statements of people who really don’t understand the science of forest management…like, “You’ve got to be kidding me, you're going to harvest and right after harvest you're going to light it on fire? I mean, what more can you do to the land to punish it?” They just don’t understand how mechanical treatment followed up by prescribed fire is probably going to get you the best results for the fuel reduction…it takes patience to allow people at different stages in their understanding to develop a better awareness of what we’re all trying to accomplish.

Interviewees suggested that residents “don’t understand” agency goals, believed that mitigation meant cutting down all of the trees, and worried that projects would reduce privacy. Both state and federal agency employees also argued that the public is not “accepting of smoke in the air” and described the challenge of “getting the public to understand, yes, it’s smoke now, but to prevent catastrophe later.” In response to these challenges, the Crosswalk proposes increasing public acceptance of prescribed fire and improving public understanding of the role of fire (Appendix C-4) and timely educational messages to communities.

### Agency-Agency Relationships: inadequate organizational capacity to work across boundaries

While *The Usual Suspects* focuses on barriers that are external to the agencies, including lack of funding, policy requirements, litigation, and lack of public understanding, *Agency-Agency Relationships* emphasizes barriers that are internal, including agency structure and culture, and relationships between agencies. According to this framing, the key barriers to scaling up mitigation are associated with working across organizational and jurisdictional boundaries, including the following: (1) different agencies have different missions and approaches, which can make it difficult to work together across boundaries; (2) agencies need to invest time to navigate these differences and build partnerships and trust, but they often do not have the capacity to do so; and (3) staff turnover limits agency ability to build the relationships necessary to work at larger scales across jurisdictional boundaries.

According to this Washington state agency employee, agencies “work differently…because of our missions” and not because of different policies or procedures. A Forest Service employee in Utah similarly argued that “different agencies have different missions” which leads to different approaches to wildland fire. For example, both the Crosswalk (Appendix E5) and our interviewees characterized the Forest Service as more focused on reducing risk in the wildland-urban interface as compared with the Department of Interior agencies. Interviewees also suggested that, in some regions, the Bureau of Land Management is more focused on protecting sage grouse habitat as compared with other agencies. Diverging missions and the locations of public lands influence these different approaches to wildfire.

In Washington, a local fire chief suggested they “just don’t have the same vision” as the Forest Service. They argued that local fire departments are more focused on protecting infrastructure and think about their management in terms of a “circle that radiates from the structures out.” In contrast, they characterized the Forest Service as focusing on “a plot of land that’s maybe four or five miles from a structure.” A Utah Forest Service official contrasted a state agency’s mission to “put the fire out as quick as possible” with the Forest Service desire “to manage the fire to achieve an outcome.” These examples illustrate that while the agencies may share a goal of reducing wildfire risk, how that translates to specific actions on the landscape can look very different.

Even within an agency, differences across units can challenge efforts to work together. As this interviewee points out:

There’s a different mindset to first responders, and that kind of preparedness and response mindset or mantra doesn’t always jive with what the rest of the organization in the Forest Service has going, who are, perhaps biologists who are managing a piece of ground for that particular interest, that particular program.

Thus, agency-agency partnerships may need to navigate differences within as well as across agencies.

Different terminology can also inhibit collaboration. As this County Fire Marshall from Washington described:

Mid-summer, they [USFS] put out bulletins about going to stage two for fire designation. It just threw a lot of us for a loop because we just didn’t have a clue what they were talking about initially…It was done without any consultation of any of the folks here. It was like the gorilla in the room got their way right away.

This individual also references the power dynamic when large federal agencies are perceived as calling the shots and county agencies have to catch up.

In addition to different missions, different approaches to wildland fire, and different terminology, agency staff indicated that they were focused on their own lands and jurisdictions and reluctant to tell each other how to operate. One Forest Service employee in Washington suggested that one of the “biggest” barriers was the “attitude” of the Forest Service, which they claimed has a myopic focus on their own lands. They stated:

You can imagine that over many years now the Forest Service [is] used to doing things themselves, right? We rarely go ask people for help or work across boundaries. We just have a long history of taking care of the land that we’re responsible to manage and I would say that’s generally true for states as well…That’s just the way we’ve always done it…And it’s time to try something new. A different way of working together and that just overcoming [what] I refer to as having an attitude.

This emphasis on autonomy was echoed by states and tribes as well. One interviewee who works on interagency efforts at the national-level explained that “our intent is not to tell one another how we're going to operate.” A tribal forest manager described the tribe as “individual autonomous land owners” with “sovereignty” that means that federal and state agencies cannot dictate how fire is managed on tribal lands.

Because the *Agency-Agency Relationships* framing defined the problem in terms of barriers to agencies working together, this framing also emphasized the need to build relationships across different agencies and approaches. One community wildfire leader in Washington argued that “it’s less even a matter of the policy and the mandates…it’s about relationships too.” They suggested that to “work together more effectively…that’s a relationship issue, not necessarily always a policy or goal issue.” According to this Fire Chief from Washington:

When we have our meetings, BLM comes to the meetings, Forest Service comes to the

meeting, Parks comes to the meetings. So we do face time when there’s not an emergency. When an emergency breaks out there’s a little bit more of a comfort feeling…with somebody you break bread with. You know who they are…You establish a trust with that person and that’s really how we make things work here.

While this comment focuses on responding to a fire, many interviewees pointed out that these kinds of relationships would benefit mitigation work as well. Interviewees also indicated that the incident command structure that makes collaboration work for fire response can impede collaboration on mitigation, because the “operational mindset of follow the rules, do what you're told, don’t ask questions” can limit the innovation required for scaling up mitigation.

Given the need to build relationships that enable agencies to work together, some interviewees focused on increased capacity and resources. But rather than capacity and resources to expand fuel treatment to additional areas, they argued for capacity to build relationships and collaborate, again in order to work across different agency missions and approaches. As one interviewee put it, “collaboration moves at the speed of trust.” According to an interviewee from the local government in Washington:

Probably one of the biggest barriers is just time. In coordinating our communications so that we’re on the same page…it takes a lot of time to go and build a relationship with someone else. I could work in complete isolation, and stay busy every day of the week, working just within my jurisdiction, just for my residents, without any partnerships whatsoever. And so it takes us being proactive, and making time for partnerships. And a lot of people just don’t have it. I think capacity is a big issue. People’s plates are full, and they have to prioritize their jurisdiction, and their work.

A Utah Fire Chief confirmed this challenge, describing that building relationships with the Forest Service was “not my number one focus” due to lack of capacity. Some interviewees, including this Utah state agency employee, suggested that they needed “more consistent participation” and a “firmer commitment” by federal agencies as well as “federal agency leadership” “at the table” and “engaged in discussions to help prioritize where we want to allocate resources.” A local agency employee from Washington echoed this sentiment, sharing that some federal agencies do not “engage local practitioners” and do not show up to planning meetings, saying “we just don’t see it” and “when asked, we often get no response.” This interviewee went on to suggest that a lack of alignment between agency goals might contribute to this lack of engagement, wondering if perhaps “our priorities and [our] objectives [do not] meet theirs to where they should be at the table.” In other words, key partners need to invest time to develop shared priorities, which requires navigating the different missions and approaches described above.

Getting the “right people at these conversations” was seen as a priority by many interviewees. At the same time, a tribal forest manager wondered if participation was worth the “time and effort,” especially if they are “seeing a lack of results or maybe things are moving too slowly” and there are things they “could be doing back home that would make more of an impact.” Some interviewees suggested that different agency priorities made it difficult to turn collaboration into on-the-ground projects. A local fire district employee explained that, “we collaborate really well on these ideas, but it’s getting the work on the ground that’s the disconnect.”

In this context, personnel practices and position descriptions were regarded as part of the solution. In some cases, local Fire Chiefs could not identify a particular person within the relevant federal agency whose job included working with local fire districts. As this Utah Fire Chief describes:

Our areas border one another yet we don’t have that discussion between our local jurisdiction and the federal level…we don’t have the working relationship…or even the contact… to get on the same page necessarily, or to understand one another’s goals and the barriers that do stand in the way to achieve the goals. I would love to understand better why the Forest Service has the rules they do in place…we could try to figure out a way around that, a way to address the problem in a different way.

One Utah state agency employee described the Shared Stewardship Strategy, a Forest Service initiative to partner with the states on wildfire, by saying that “it’s kind of forced collaboration that I don’t think they [the Forest Service] have time for right now.” In a time and resource-limited environment, individuals and agencies might understandably focus on what they perceive to be their core areas of responsibility. When this occurs, “partnerships and collaborations outside, that kind of takes a second seat,” according to a local fire district employee.

Many interviewees argued that one of the key barriers to building effective partnerships between agencies was staff turnover. As one interviewee explained, “the challenges we have with retention, keeping trained, qualified people here…[we’re] losing institutional knowledge at every level.” Frequent employee reassignment and turnover in federal agencies, particularly in the Forest Service, has been recognized as problematic (Davenport et al. 2007). While routine reassignment can create a workforce with broad experience in a variety of contexts, interviewees argued that turnover can also compromise relationships with partners. A tribal land manager explained how they “struggle” with staff turnover in the federal agencies, saying:

It seems like it’s a new face every year. So I have to build up that working relationship…We have some agreements in place. But it is a bit of a learning process…people that you’re used to working with, you built a working relationship, there’s some comfort there, there’s some confidence there, some trust. And you have to rebuild that with someone new every 18 months or so. That becomes problematic. And I just don’t think we move as fast as we could…But it’s places that you have a long tenured ranger or a long tenured forest supervisor who has a good working relationship with the tribe. And they’ve done some good things when those occurrences exist.

According to this interviewee, long-term working relationships enable on-the-ground, landscape-scale mitigation. A state agency employee in Utah talked about the “cyclical” nature of these relationships, saying that “all it takes is one line officer, somebody in leadership, the next FMO [Fire Management Officer] forester who doesn't buy into these types of cooperative partnership approaches. And it could make things really difficult again.”

A local-level wildfire professional in Washington also talked about the problem of building trust with “migratory staff within the agency,” drawing a direct link between turnover and challenges in managing risk. According to this interviewee, “in terms of co-managing risk on the landscape, if you think you’re getting to know the person, you’re trying to collectively come to some understandings about how to work on the landscape. Then the person leaves.” Similar to the tribal land manager above, this interviewee connects the ability to do on-the-ground work with relationships that are enabled by long-term tenure in particular positions. Interviewees in Utah described identical challenges, explaining that “a lot of turnover” and “churning” meant “constantly” rebuilding relationships and also directly connected these barriers to “this question of shared risk.” This framing illustrates the ways in which agencies themselves are creating barriers through the formal institutional structures that move staff frequently, and through a culture that de-prioritizes the time-consuming activities of engaging in collaborative work.

### Engaging the Public: barriers to meaningful public engagement

According to *The Usual Suspects*, policy requirements and public engagement are regarded as barriers that needed to be streamlined and limited. However, in the *Engaging the Public* framing, litigation and lengthy NEPA processes are regarded as a symptom of the problem, which is viewed as a lack of meaningful public engagement. Thus, more robust public engagement and collaboration are advanced as the solution.

Interviewees who focused on this framing emphasized the need for multi-stakeholder collaboration. According to this Utah state agency employee:

Having these work groups in place, having it be a safe place where people can talk about fire, and wildfire risk reduction, making sure that the broader partners know one another, so it’s not just Forest Service, BLM, and state fire staff talking to one another. There’s more participation and input.

The Crosswalk also envisions collaborative groups as an effective mechanism to build broad support for mitigation work, stating that “collaborative groups have proven to be extremely successful in developing consensus in regard to federal fuel reduction projects” (Appendix F7-F8). Some interviewees suggested that collaboration could reduce litigation and the delays that often accompany lawsuits. As this Regional-level Forest Service employee suggests, “collaboration builds a common understanding and builds relationships that increase social license and social capacity…successful collaboration in some places means less litigation.” A senior Forest Service employee in Washington concurred, saying:

I think the risk is not collaborating…it’s the hardest form of decision making you can have. It’s very time consuming…if we design into a project, it’s going to take some work and discussion to try to get our collaborative to support it…The alternative is not collaborating, and buzzing through the early part real fast, and then bogging it down in objections in court at the end. It’s one or the other.

In other words, they are suggesting that streamlining the initial planning process could increase the risk of litigation.

Interviewees also suggested that earlier public engagement might reduce lawsuits. According to a Regional-level Forest Service employee, the “agency needs to do a lot more work pre-NEPA to work with public and stakeholders to figure out what you agree on, so that by the time you do NEPA you don’t get sued.” Interviewees also argued that fear of litigation meant that federal agencies spent more time on NEPA than required. These interviewees contested the claim that NEPA needs to be streamlined, suggesting instead that NEPA is “not broken” and “not the barrier they think it is,” but that “fear of lawsuits” was motivating lengthy and cumbersome NEPA processes.

Collaboration was seen as a way for agencies to better understand the needs of the public as well as a way to change public attitudes. This Washington state employee explained the connection between policy, public attitudes toward mitigation, and collaboration in this way:

So in order to make that change you’re going to have to change rules and regulations. But primarily, you’re going to have to change the attitude of the public. And the only way you’re going to do that is to get a collaboration of different organizations and individuals that are advocating for the same thing.

They are suggesting that collaboration can build a shared understanding of the problem and solutions, such that public views shift and policy change is possible. A Forest Service employee made the point that collaboration can also help agencies understand local community concerns, using this example:

This community has a very low tolerance for smoke, and the reason they have a low tolerance for smoke is that when there is smoke in the air, tourists from the west side [of the state] do not come here… the entire tourist economy grinds to almost a halt, and [in] the short period of time that these businesses have to make money, that really makes them mad.

The two-way dialogue inherent in these kinds of engagements provided opportunities to share these types of concerns and to build shared priorities.

Interviewees also acknowledged that multi-stakeholder collaboratives require substantial investments to succeed. As one NGO employee who works across the West pointed out:

It takes 10 years to develop a group of trusted stakeholders who can effectively co-manage programs, projects, multi-jurisdictional goals and objectives…you have to start by creating a group of stakeholders that form into a collaborative, that have a couple of leaders who can back each other up, and have a sustainable model, where these folks achieve incremental accomplishments annually, and develop momentum.

These investments were believed to pay off in the long-term as collaboratives built momentum through successful projects and expanded the scale of their mitigation work.

Similar to *Agency-Agency Relationships*, this framing also emphasizes the lack of agency capacity to engage, but in this case the focus was on the need for engagement with multi-stakeholder collaboratives and with the public more broadly. One community leader described the impact of this lack of capacity, saying “I don’t think they have enough money to do a damn thing…there’s not enough people to do the work that they need to do. I have reached out to the Forest Service on a number of occasions and I've not even got a phone call back.” A local NGO-employee shared a similar sentiment, suggesting that “they are under water here. On the National Forest there is no capacity remaining to engage in the community…Their budgets are small. They’re overworked and understaffed.” This interviewee also explicitly connected a lack of engagement with job descriptions and capacity, saying “It’s nobody’s job and nobody has time…There’s just no one around this table because they don’t think they have the time or the capacity.” They went on to suggest that agencies invest in collaborative capacity and specifically positions that focus on coordination, suggesting that “Other ways would be to actually invest in our people, our collaborative and coordinating capacity. Jobs like…don't exist because they are hard to fund, and they are even harder to convince an agency that it's worthwhile.” Thus, lack of capacity does not simply hinder the ability of the agencies to plan and implement fuel reduction projects, it also limits the ability of the agencies to effectively engage the public. Again, echoing the Agency-Agency Relationships frame, high rates of agency staff turnover were believed to compromise collaboration, especially given how long it takes to develop a successful collaboration.

## Discussion

As Bardwell (1991) aptly put it, “there are many ways of looking at the problem, many paths worth exploring, and rarely is there one ‘right’ solution.” Burns and Cheng (2007) caution against the assumption that there is agreement on wildfire problem frames. However, familiar mental maps and biases toward existing assumptions make it difficult to see past favored problem framings (Bardwell 1991). Frames can be quite stable and even “locked in” (Elliott et at. 2003). Further, individuals often only pay attention to one particular way of framing a problem and take for granted the assumptions that underlie that framing (Cairney and Kwiatkowski 2017). But when people develop new ways of understanding an issue, they can shift frames and reveal new ways of solving a problem (Gray 2003). Reframing or redefining a problem can help people examine the “problem space” in ways that expand the range of solutions that they consider (Bardwell 1991). Reframing requires reflecting on the different ways of viewing a particular problem (Elliott et al. 2003).

In this study, we examine three different ways of framing the wildfire problem (see Table 2). Each of these frames privileges the solutions that align with a particular problem definition, and places blame and responsibility on different actors. The frames legitimize and prioritize some solutions, while devaluing and excluding others.

**Table 2:** Barriers and investments advanced by different framings

**Framing**	**Barriers**	**Investments/solutions**
The Usual Suspects^a^	Lack of capacity/funding	Increase resources for fuel treatment Develop mechanisms to share resources
	Procedural delays	Streamline NEPA/policy requirements Limit public engagement
	Delays due to litigation	Limit litigation
	Lack of shared understanding	Educate the public
Agency-Agency Partnerships^b^	Different agency missions make it difficult to scale up to landscape-level	Increase agency capacity to partner and negotiate across agency differences to design and implement cross-boundary projects Promote relevant skills through position descriptions, training, and evaluation
	Lack of capacity to partner	
	Turnover compromises relationships	Rethink career ladders and promotion systems
Engaging the Public^b^	Lack of meaningful public engagement	Invest in forums for deliberation, including pre-NEPA collaboration and NEPA public engagement to build shared understandings and public support, and reduce risk of litigation Promote relevant skills through position descriptions, training, and evaluation
	Lack of capacity to engage public	
	Turnover compromises relationships	Rethink career ladders and promotion systems

^a^Most of *The Usual Suspects* solutions require action on the part of Congress and other external actors

^b^Note the similarities in *Agency-Agency Partnerships* and *Engaging the Public* solutions, most of which could be accomplished through internal changes to agency structure and culture that emphasize public engagement, partnerships, and collaboration

*The Usual Suspects* focused almost entirely on barriers that are imposed by actors outside of the federal land management agencies, barriers such as lack of adequate resources, cumbersome policy and procedural requirements, delays due to litigation, and lack of public understanding. The solutions—increase funding, streamline NEPA, limit litigation, and educate the public—rest on the assumption that the agencies need more resources and fewer restrictions to develop and implement mitigation projects at a faster pace. The problem was defined as external to the federal agencies and thus solutions often depend on other actors, from Congress to the public.

If the problem was framed in terms of the need for agencies to collaborate across jurisdictional boundaries, as in *Agency-Agency Partnerships*, organizational barriers were privileged. Here barriers were related to the ways that agencies are structured, from position descriptions to promotional systems, and how those structures constrain the development of effective cross-boundary wildfire work and the scaling-up of mitigation. The assumption here is that scaling up requires that agencies work together, to both increase the spatial scale of mitigation and to build on each other’s strengths. As a result, the solutions were largely internal to the agencies, focused on the need for increased capacity to navigate different agency missions and approaches, and to build agency-agency partnerships and trust. These findings align with previous research documenting that different agency goals can limit cross-jurisdictional wildfire work (Meyer et al. 2015), that agencies need to proactively address conflicting mandates and priorities (Flemming et al. 2015), that managers view collaborative forums and federal-state agency relations as key to addressing barriers in particular places (Schultz et al 2019a), and that strong partnerships can promote collective action to address wildfire risk across jurisdictional boundaries (Charnley et al. 2020). Since agencies cannot typically compel one another to act, they need the capacity to negotiate a shared understanding of the problem and to co-develop priorities and work plans. Based on this framing, potential investments include building agency capacity to partner; addressing staff turnover by rethinking career ladders and promotion systems; and promoting relevant skill sets through position descriptions, trainings, and performance evaluations that emphasize collaborative and partnering skills.

The *Engaging the Public* framing shifts the focus toward collaboration beyond the agencies, and barriers that limit public engagement and multi-stakeholder collaboration. Here, lack of public support for scaling up mitigation is assumed to be a result of limited opportunities to develop a shared understanding of the problem and to collaborate on wildfire mitigation. The solution then is to invest in forums for deliberation and mutual learning, early on during pre-NEPA phases as well as through the NEPA process. The assumption here is quite different from *The Usual Suspects*, which advances education as a way to build public support, suggesting there is a correct way of thinking about wildland fire and mitigation work. In contrast, *Engaging the Public* assumes that shared understandings are negotiated and developed through dialogue, and thus the need for meaningful public engagement and collaboration. Again, this is in contrast to *The Usual Suspects*, which argues that NEPA “encourages too much engagement” and that planning processes should be expedited. Further, litigation is not viewed as a barrier in the *Engaging the Public* framing, but rather as a symptom of the problem. In the absence of meaningful ways for members of the public to engage with decision-making, the only recourse is to litigate the projects that they oppose. The assumption here is that early, meaningful public engagement and collaboration can reduce litigation and the delays that often accompany lawsuits.

To address the challenges of competing problem definitions, Schultz et al. (2019b) recommend a “meta-frame” that integrates across different ways of framing the problem. Perhaps the most important synergy across the frames is the need for building collaborative capacity to share resources and enable agency-agency partnerships, meaningful public engagement, and multi-stakeholder collaboratives. Sturtevant et al. (2005, p. 29) identified the critical need to build capacity for collaboration back in 2005, stating that “the Forest Service is chronically short of funding for collaborative efforts, and what funding is available is inconsistent” and specifically citing the need for dedicated staff time and incentives in performance evaluations. Schultz and Moseley (2019) suggest a number of ways to build collaborative capacity, including positions dedicated to coordinating across land management agencies and air quality regulators to advance prescribed burning; federal grants for states, tribes, and communities to pursue locally appropriate solutions; state funding for air quality monitoring and permitting; and staff specifically assigned to collaborations. They also point out that “approaches that invest in places where collaboration exists also may leave behind communities without capacity, making it critical to address how to build capacity where it does not already exist” (Schultz and Moseley 2019:39). Thus, meaningful efforts to build collaborative capacity need to focus on a suite of actors, including but not limited to federal agencies, as well as communities with different types of capacity. Further, recent research on wildland fire recommends stakeholder engagement, multi-stakeholder processes, strong partnerships, and increased deliberation (Charnley et al. 2020, Schultz et al. 2019b, Wilson et al. 2018), suggesting that these processes influence which solutions are “politically possible” (Wilson et al. 2018).

Investing in collaborative capacity requires the recognition that building partnerships, shared understandings, and public support take time and resources. And because of the time and investment required, collaborative work is often depicted as in tension with the urgent need to scale up mitigation. As such, the different frames described above could be characterized as competing, given limited resources, and the assumption that partnerships and collaboration take longer than more traditional unilateral approaches to planning. This sense of competition is exacerbated by the urgency of the wildfire problem or “crisis,” and the need to address fire risk and scale up mitigation as quickly as possible. In the context of this urgency, instead of carefully examining the problem and the suite of potential solutions, the dominant frame may prevail and constrain investment to a narrow set of actions, which might fail to address all of the relevant barriers.

Further, the scale, scope, and urgency of a worsening wildfire problem means that while solutions like streamlining NEPA and reducing litigation are incredibly tempting, they may compromise public support and increase litigation, constraining efforts to scale up mitigation. As the agencies attempt to scale up mitigation, the appropriate role of litigation will likely continue to be debated. Ostergren et al. (2006:380) characterizes the debate around appeals and litigation, public engagement, and agency decision-making as “particularly fierce” and argues for “more accessible, less legalistic avenues” for public engagement. However, litigation and appeals provide the public with mechanisms to hold the agencies accountable and ensure that they follow policy requirements intended to protect ecosystems and public health. Thus, the option to litigate may provide an important avenue to contest proposals in a democratic system. A recent study in Idaho by McIver and Becker (2021) found that collaborative projects were more likely to be litigated, which contradicts claims made by our interviewees that collaboration reduces litigation, pointing to the need for additional research on the relationship between collaboration and litigation.

If investments in collaborative capacity help agencies to scale up mitigation across jurisdictional boundaries and build projects that have broad public support, then perhaps slowing down in the short-term to invest in collaboration will ultimately enable a long-run increase in the pace and scale of on-the-ground mitigation work. In other words, if the agencies play the long game and invest in collaborative capacity, they might be able to *go slow to go fast*. Taking time to build a shared vision across diverse partners and publics may ultimately result in more on-the-ground fuel reduction, more effectively scaling up mitigation work over the medium to long-term. However, the assumption that partnering and collaborating delays on-the-ground work may itself be flawed. McIver and Becker (2021) found that collaborative projects in Idaho treated more on-the-ground acres, realized a more diverse set of objectives, and were accomplished with more efficiency, as compared with more traditional projects. McIver and Becker (2021) argue that their research “dispels conventional wisdom that land management agencies must sacrifice efficiency for greater public involvement.” These findings are promising, but additional research is needed to determine when, where, and how investments in collaborative capacity influence the pace and scale of on-the-ground mitigation.

But perhaps the focus on the pace and scale of mitigation obscures a more fundamental contradiction between the frames. *The Usual Suspects* envisions wildfire as a technical problem with solutions that are mechanical and biophysical, emphasizing on-the-ground fuel reduction. When we reframe and focus on the need for partnerships and collaboration, the wildfire problem is also a social and political problem that requires wading into the messy and unpredictable world of communities, organizations, and public debate. If all of these frames are to some extent “correct,” and simply privilege different aspects of the problem, then we need to understand and invest in the most effective solutions to address social and political barriers, just as we need to understand and invest in the most effective ways to reduce fuels and address biophysical fire risk.

## Conclusion

Agencies have limited resources to invest, millions of acres where wildfire risk needs to be addressed, and a lack of clarity regarding how to prioritize investments. Reframing the wildfire problem through careful analysis of different problem definitions can illuminate why some solutions are favored over others and how investments might better address the full range of barriers. Although funding for wildfire mitigation is being expanded through efforts such as the recently unveiled 10-year plan, the scope of mitigation is still daunting. Beyond funding to treat additional acres, investments could target changes that build agency capacity for public engagement, effective partnerships, and multi-stakeholder collaboration, which would increase the reach and impact of mitigation.

## Data Availability

Raw data is not available because it contains information that could compromise research participant privacy/consent.

## References

[CR1] Abatzoglou JT, Williams AP (2016). Impact of anthropogenic climate change on wildfire across western US forests. Proceedings of the National Academy of Sciences USA.

[CR2] Bardwell LV (1991). Problem-framing: A perspective on environmental problem-solving. Environmental Management.

[CR3] Bowman DMJS, Kolden CA, Abatzoglou JT, Johnston FH, van der Werf GR, Flannigan M (2020). Vegetation fires in the Anthropocene. Nature Reviews Earth and Environment.

[CR4] Brandenburg AM, Carroll MS (1995). Your place or mine? The effect of place creation on environmental values and landscape meanings. Society and Natural Resources.

[CR5] Brenkert-Smith H (2010). Building bridges to fight fire: The role of informal social interactions in six Colorado wildland–urban interface communities. International Journal of Wildland Fire.

[CR6] Brenkert-Smith, H, JR Meldrum, PA Champ, and CM Barth. 2017. Where you stand depends on where you sit: qualitative inquiry into notions of fire adaptation. Ecology and Society 22(3). 10.5751/ES-09471-220307

[CR7] Brenkert-Smith H, Jahn JLS, Vance EA, Ahumada J (2019). Resistance and representation in a wildland–urban interface fuels treatment conflict: The case of the Forsythe II Project in the Arapaho-Roosevelt National Forest. Fire.

[CR8] Brunson MW, Shindler BA (2004). Geographic variation in social acceptability of wildland fuels management in the Western United States. Society and Natural Resources.

[CR9] Burke, M, A Driscoll, J Xue, S Heft-Neal, J Burney, and M Wara. 2020. The changing risk and burden of wildfire in the US. National Bureau of Economic Research Working Paper No. 27423. http://www.nber.org/papers/w27423

[CR10] Burns M, Cheng AS (2007). Framing the need for active management for wildfire mitigation and forest restoration. Society and Natural Resources.

[CR11] Cairney P, Kwiatkowski R (2017). How to communicate effectively with policymakers: Combine insights from psychology and policy studies. Palgrave Communications.

[CR12] Charnley S, Poe MR, Ager AA, Spies TA, Platt EK (2015). A burning problem: Social dynamics of disaster risk reduction through wildfire mitigation. Human Organization.

[CR13] Charnley S, Kelly EC, Fischer P (2020). Fostering collective action to reduce wildfire risk across property boundaries in the American West. Environmental Research Letters.

[CR14] Cleaves DA, Martinez J, Haines TK (2000). Influences on prescribed burning activity and costs in the national forest system. Gen. Tech. Rep. SRS-37.

[CR15] Collins K, Ison R (2009). Living with environmental change: Adaptation as social learning. Environmental Policy and Governance.

[CR16] Crow AD, Lawhon LA, Berggren J, Huda J, Koebele EA, Kroepsch A (2017). A narrative policy framework analysis of wildfire policy discussions in two Colorado communities. Politics and Policy.

[CR17] Crow AD, Berggren J, Lawhon LA, Koebele EA, Kroepsch A, Huda J (2017). Local media coverage of wildfire disasters: An analysis of problems and solutions in policy narratives. Environment and Planning C: Politics and Space.

[CR18] Davenport MA, Anderson DH, Leahy JE, Jakes PJ (2007). Reflections from USDA forest service employees on institutional constraints to engaging and serving their local communities. Journal of Forestry.

[CR19] Diaz JM, Steelman T, Nowell B (2016). Local ecological knowledge and fire management: What does the public understand?. Journal of Forestry.

[CR20] Druckman JN (2001). The implications of framing effects for citizen competence. Political Behavior.

[CR21] Dupéy LN, Smith JW (2018). An integrative review of empirical research on perceptions and behaviors related to prescribed burning and wildfire in the United States. Environmental Management.

[CR22] Elliott M, Gray B, Lewicki RJ, Lewicki RJ, Gray B, Elliott M (2003). Lessons learned about the framing and reframing of intractable environmental conflicts. Making Sense of Intractable Environmental Conflicts: Concepts and Cases.

[CR23] Flemming CJ, McCartha EB, Steelman TA (2015). Conflict and collaboration in wildfire management: The role of mission alignment. Public Administration Review.

[CR24] Gabbert, B. 2022. Forest Service announces 10-year initiative to increase fuel treatment. Wildfire Today. https://wildfiretoday.com/2022/01/19/forest-service-announces-10-year-initiative-to-increase-fuel-treatment/

[CR25] Gray B, Lewicki RJ, Gray B, Elliott M (2003). Framing environmental disputes. Making Sense of Intractable Environmental Conflicts: Concepts and Cases.

[CR26] Hesse-Biber SN, Leavy P (2006). The practice of qualitative research.

[CR27] Jasanoff S, Wynne B, Rayner S, Malone EL (1998). Science and decisionmaking. Human choices and climate change.

[CR28] Klyza CM (1991). Framing the debate in public lands politics. Policy Studies Journal.

[CR29] Kolden CA (2019). We’re not doing enough prescribed fire in the Western United States to mitigate wildfire risk. Fire.

[CR30] Layder D (1998). Sociological practice: Linking theory and social research.

[CR31] McCaffrey SM (2004). Fighting fire with education: What is the best way to reach out to homeowners?. Journal of Forestry.

[CR32] McCaffrey S, Toman E, Stidman M, Shindler B (2013). Social science research related to wildfire management: An overview of recent findings and future research needs. International Journal of Wildland Fire.

[CR33] McCaffrey S.M., Olsen C.S. (2012). Research perspectives on the public and fire management: a synthesis of current social science on eight essential questions. General Technical Report NRS-GTR-104.

[CR34] McIver CP, Becker DR (2021). An empirical evaluation of the impact of collaboration on the pace and scale of National Forest management in Idaho. Forest Science.

[CR35] Meyer MD, Roberts SL, Wills R, Brooks M, Winford EM (2015). Principles of effective USA federal fire management plans. Fire Ecology.

[CR36] Miller BA, Yung L, Wyborn C, Essen M, Gray B, Williams DR (2022). Re-envisioning wildland fire governance: addressing the transboundary, uncertain, and contested aspects of wildfire. Fire.

[CR37] Mylek MR, Schirmer J (2020). Understanding acceptability of fuel management to reduce wildfire risk: Informing communication through understanding complexity of thinking. Forest Policy and Economics.

[CR38] National Strategic Committee. 2016. The Cohesive Strategy Crosswalk and Strategic Alignment Report and Appendices. https://www.forestsandrangelands.gov/documents/strategy/reports/cohesive_strategy_crosswalk_and_strategic_alignment_report.pdf. https://www.forestsandrangelands.gov/documents/strategy/reports/cohesive_strategy_crosswalk_and_strategic_alignment_appendices.pdf

[CR39] Nielsen-Pincus M, Ager A, Evers C (2019). Exposure complexity and community capacity to manage wildfire risk: A coupled biophysical and sociological analysis of 60 communities in the Western United States. Fire.

[CR40] Nisbet MC (2009). Communicating climate change: Why frames matter for public engagement. Environment: Science and Policy for Sustainable Development.

[CR41] Olsen CS, Sharp E (2013). Building community–agency trust in fire-affected communities in Australia and the United States. International Journal of Wildland Fire.

[CR42] Olsen CS, Shindler BA (2010). Trust, acceptance, and citizen–agency interactions after large fires: Influences on planning processes. International Journal of Wildland Fire.

[CR43] Olsen CS, Toman E, Frederick S (2017). A multi-region analysis of factors that influence public acceptance of smoke from different fire sources. International Journal of Wildland Fire.

[CR44] Ostergren DM, Lowe KA, Abrams JB, Ruther EJ (2006). Public perceptions of forest management in North Central Arizona: The paradox of demanding more involvement but allowing limits to legal action. Journal of Forestry.

[CR45] Patterson ME, Williams DR (2002). Collecting and analyzing qualitative data: Hermeneutic principles, methods, and case examples.

[CR46] Quinn-Davidson LN, Varner JM (2012). Impediments to prescribed fire across agency, landscape and manager: an example from northern California. International Journal of Wildland Fire.

[CR47] Roman, J, A Verzoni, and S Sutherland. 2020. Greetings from the 2020 wildfire season: Five undeniable truths from a pivotal year in the world's growing struggle with wildfire. NFPA Journal: The Magazine of the National Fire Protection Association. https://www.nfpa.org/News-and-Research/Publications-and-media/NFPA-Journal/2020/November-December-2020/Features/Wildfire

[CR48] Rubin HJ, Rubin IS (2005). Qualitative interviewing: The art of hearing data.

[CR49] Schultz CA, Moseley C (2019). Collaborations and capacities to transform fire management. Science.

[CR50] Schultz CA, McCaffrey SM, Huber-Stearns HR (2019). Policy barriers and opportunities for prescribed fire application in the western United States. International Journal of Wildland Fire.

[CR51] Schultz CA, Thompson MP, McCaffrey SM (2019). Forest Service fire management and the elusiveness of change. Fire Ecology.

[CR52] Stone D (1989). Policy Paradox: The Art of Political Decision Making.

[CR53] Sturtevant V, Moote MA, Jakes P, Cheng AS (2005). Social science to improve fuels management: A synthesis of research on collaboration. Gen. Tech. Rep. NC-257.

[CR54] The National Strategy: The Final Phase in the Development of the National Cohesive Wildland Fire Management Strategy*. 2014.* https://www.forestsandrangelands.gov/documents/strategy/strategy/CSPhaseIIINationalStrategyApr2014.pdf

[CR55] Toman E, Stidman M, Shindler B, McCaffrey S (2011). Reducing fuels in the wildland–urban interface: Community perceptions of agency fuels treatments. International Journal of Wildland Fire.

[CR56] Toman E, Stidham M, McCaffrey S, Shindler B (2013). Social science at the wildland-urban interface: A compendium of research results to create fire-adapted communities. Gen. Tech. Rep. NRS-111.

[CR57] Toman E, Shindler B, McCaffrey S, Bennett J (2014). Public acceptance of wildland fire and fuel management: Panel responses in seven locations. Environmental Management.

[CR58] USDA Forest Service. 2015. The rising cost of wildfire operations: Effects on the Forest Service’s non-fire work. Washington, D.C. USA. https://www.fs.usda.gov/sites/default/files/2015-Fire-Budget-Report.pdf

[CR59] USDA Forest Service. 2022. Confronting the wildfire crisis: A 10-year implementation plan. Report FS–1187b. https://www.fs.usda.gov/sites/default/files/Wildfire-Crisis-Implementation-Plan.pdf

[CR60] Vaske JJ, Absher JD, Bright AD (2007). Salient value similarity, social trust, and attitudes toward wildland fire management strategies. Human Ecology Review.

[CR61] Williams DR, Jakes PJ, Burns S, Cheng AS, Nelson KC, Sturtevant V, Brummel RF, Staychock E, Souter SG (2012). Community wildfire protection planning: The importance of framing, scale, and building sustainable capacity. Journal of Forestry.

[CR62] Wilson PI, Paveglio T, Becker D (2018). The politically possible and wildland fire research. Fire.

[CR63] Yung L, Patterson M, Freimund WA (2010). Rural community views on the role of local and extra-local interests in public lands governance. Society and Natural Resources.

[CR64] Yung L, Schwarze S, Carr W, Chapin FS, Marris E, Hobbs RJ, Higgs ES, Hall CM (2013). Engaging the public in novel ecosystems. Novel Ecosystems: Intervening in the New Ecological World Order.

